# Wilson’s Disease: Facing the Challenge of Diagnosing a Rare Disease

**DOI:** 10.3390/biomedicines9091100

**Published:** 2021-08-28

**Authors:** Ana Sánchez-Monteagudo, Edna Ripollés, Marina Berenguer, Carmen Espinós

**Affiliations:** 1 Rare Neurodegenerative Diseases Laboratory, Centro de Investigación Príncipe Felipe (CIPF), 46012 Valencia, Spain; asanchez@cipf.es (A.S.-M.); eripolles@cipf.es (E.R.); 2 Joint Unit on Rare Diseases CIPF-IIS La Fe, 46012 Valencia, Spain; marina.berenguer@uv.es; 3 Hepatology-Liver Transplantation Unit, Digestive Medicine Service, IIS La Fe and CIBER-EHD, Hospital Universitari i Politècnic La Fe, 46026 Valencia, Spain; 4 Department of Medicine, Universitat de València, 46010 Valencia, Spain; 5 Centro de Investigación Biomédica en Red de Enfermedades Hepáticas y Digestivas, CIBERehd, Instituto de Salud Carlos III, 28029 Madrid, Spain

**Keywords:** Wilson’s disease, Leipzig scale, *ATP7B* gene, Wilson-like, genetic modifiers, biomarkers

## Abstract

Wilson disease (WD) is a rare disorder caused by mutations in *ATP7B*, which leads to the defective biliary excretion of copper. The subsequent gradual accumulation of copper in different organs produces an extremely variable clinical picture, which comprises hepatic, neurological psychiatric, ophthalmological, and other disturbances. WD has a specific treatment, so that early diagnosis is crucial to avoid disease progression and its devastating consequences. The clinical diagnosis is based on the Leipzig score, which considers clinical, histological, biochemical, and genetic data. However, even patients with an initial WD diagnosis based on a high Leipzig score may harbor other conditions that mimic the WD’s phenotype (Wilson-like). Many patients are diagnosed using current available methods, but others remain in an uncertain area because of bordering ceruloplasmin levels, inconclusive genetic findings and unclear phenotypes. Currently, the available biomarkers for WD are ceruloplasmin and copper in the liver or in 24 h urine, but they are not solid enough. Therefore, the characterization of biomarkers that allow us to anticipate the evolution of the disease and the monitoring of new drugs is essential to improve its diagnosis and prognosis.

## 1. Introduction

Wilson’s disease (WD; MIM 277900) is an autosomal recessive disorder related to the metabolism of copper, a metal that accumulates in tissues, mainly in the liver and brain. WD is caused by mutations in the *ATP7B* gene, which encodes for a copper transporter that is responsible for biliary excretion of copper and its incorporation into ceruloplasmin. WD was first described by the British neurologist Samuel Alexander Kinnier Wilson as a “progressive lenticular degeneration accompanied by cirrhosis in the liver” [1]. The manuscript included six cases that presented with involuntary movements, spasticity, dysarthria, psychiatric disturbances, and advanced cirrhosis. In the first half of the 20th century, several researchers made important contributions to the knowledge of the pathophysiological mechanisms of the disease. Rumpel reported excess copper in the liver of a patient with WD for the first time, and Cumings corroborated it and established that copper accumulation in the liver and in the basal ganglia were the etiological basis of the disease [2,3]. Less than 50 years after its discovery, WD became the first chronic hereditary liver disease that had a specific treatment, which made it possible to stop the devastating progression of the disease and prevent the eventual death. Despite these initial advances, its diagnosis remains a challenge and WD is currently an underdiagnosed disorder. Epidemiologic estimates have indeed established notable differences between clinical and genetic prevalence, which may be due to multiple factors including genetic modifiers, epigenetics, and habits. The main underlying problem lies in suboptimal diagnostic tests and elusive biomarkers to diagnose a rare genetic disease characterized by incomplete penetrance and highly variable expression. In this review, we discuss the difficulties in achieving a definitive diagnosis and the need for biomarkers to improve the diagnosis of WD.

## 2. Clinical Picture of Wilson’s Disease

**Figure 1:**
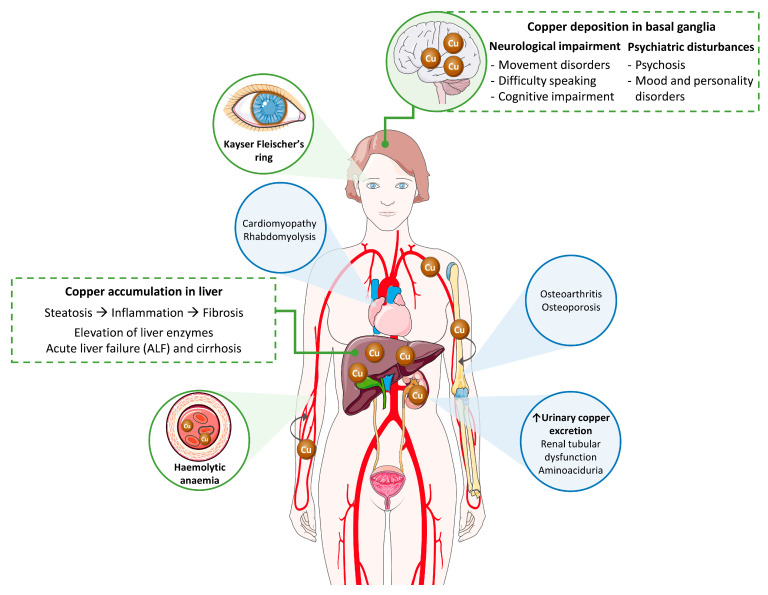
Copper toxicity in the pathogenesis of Wilson’s disease. Main signs and symptoms observed in patients are included in green boxes and circles, while secondary findings are contained in blue circles.

Wilson’s disease (WD) is a multisystemic disorder with variable symptoms characterized by abnormal deposits of copper. Clinical signs depend on the organs where copper is accumulated over the course of the pathological process (Figure 1; reviewed in [4,5,6]). Accordingly, liver and neurological involvement predominates, and WD can present as a liver, neurological or mixed disease. Most pediatric patients usually exhibit a liver presentation, whereas patients diagnosed in adulthood predominantly manifest with a mixed presentation. While diagnosis is typically made in childhood, adolescence, or early adulthood, between 5 and 35 years old, late presentations have been described.

Reasons that explain different phenotypes despite similar mutations are unclear. Interestingly, gender differences have been described in WD. In a large series with 627 patients (337 men versus 290 women), neuropsychiatric disturbances (mainly rigidity-tremor, rigidity and psychiatric symptoms) predominated and occurred in men, while hepatic presentation was more common in women [7]. Gender differences have been observed even on brain magnetic resonance imaging (MRI). With neurological presentation, men suffer from cerebellar atrophy and cortical brain atrophy more often than women, whereas women suffer from globus pallidus lesions more frequently than men; with respect to the hepatic presentation, no variability is appreciated between sexes [8]. As with many disorders, differences seem to be attributed to a protective action of estrogens that may exert antioxidant, neurotrophic, and anti-inflammatory effects, but also partly to a distinct iron metabolism between men and women [9].

### 2.1. Clinical Signs

#### 2.1.1. Liver Disease

Liver damage ranges from steatosis to chronic hepatitis, fibrosis, and cirrhosis. Clinically, it may present as asymptomatic hypertransaminasemia, with any of the manifestations of liver cirrhosis, or as severe acute liver failure (ALF). Diagnosis should be suspected when faced with patients, particularly children, adolescents, or young adults with any manifestations of liver disease without known etiological factor and/or when signs of neurological disease are also present. Of note, only 3.5% of patients with WD are diagnosed after 45 years of age, while more than half of the cases show some symptom or biochemical alteration during childhood (after 3–4 years) or in adolescence. ALF may be the initial manifestation of latent WD or may develop weeks to months after treatment non-compliance. It represents 5% of clinical presentations and is more frequent in women. Most patients have an underlying cirrhosis, and the clinical picture is difficult to distinguish from that observed in the course of viral or toxic hepatitis. Urgent liver transplantation is generally required in these cases to avoid fatal outcome. Although not always present, Coombs negative hemolytic anemia, Kayser-Fleischer (KF) ring (present in about 50%), an AST/ALT ratio > 2 and low alkaline phosphatases serum levels are findings that help reaching a diagnosis in patients with severe acute presentation. In contrast to other chronic liver conditions, liver cancer rarely develops in WD patients, yet ultrasound screening is recommended in those with cirrhosis. Differential diagnosis includes autoimmune hepatitis in children and non-alcoholic steatohepatitis in adults.

#### 2.1.2. Neurological and Psychiatric Disease

WD may have a neurological presentation. This is rarely seen in childhood but may present in adolescence or early adulthood. In adults with initial neurological presentation, liver damage, including underlying cirrhosis is typically present but may occur in the absence of any clinical symptom or alteration of liver enzymes. Low platelet count as an indirect marker of portal hypertension may be the only laboratory abnormality detected in these patients [10].

Neurological WD encompasses a wide spectrum of movement disorders caused by the degeneration of the basal ganglia (Figure 1). Most of the neurological signs consist of a movement disorder associated with bulbar symptoms [11]. The more common movement disorders are tremor, dystonia, or parkinsonism. Bulbar symptoms include dysarthria, drooling and/or dysphagia. Moreover, additional features may also happen, such as cerebellar dysfunction, chorea, hyperreflexia, seizures, restless legs syndrome, sleep disturbances, myoclonus, and cognitive impairment [5]. Recently, a semiquantitative scale for assessing brain MRI abnormalities has been validated [12] (please see Section 2.2.6). Psychiatric symptoms secondary to neurological deterioration are frequent in patients with WD. They tend to go unnoticed in the early stages of the disease, causing delays in diagnosis. In adolescents, behavioral changes (irritability, disinhibition), emotional instability, and poor academic performance are relatively common, while adult patients may show personality disorders, depression, anxiety, psychosis, and schizophrenia [13]. Two score systems have been developed and validated to help with the assessment of neurological impairment: Unified Wilson’s Disease Rating Scale (UWDRS) and Global Assessment Scale for Wilson’s Disease (GAS for WD). The UWDRS [14] consists of thirty items grouped in three subscales: neurological (27 items; 208 points), hepatic (9 items; 36 points), and psychiatric (19 items; 76 points) with a total score of 320 points. Some of the items are divided into others (i.e., tremor in arms is divided into two: postural tremor and wing-beating tremor); so that the scale actually includes the evaluation of 64 aspects, each of which is scored on an ascending five-point scale (0–4). The GAS for WD [15] comprises two tiers: global disability (4 items) and neurological assessment (14 items). Global disability covers four non-commensurable domains (liver, cognition and behavior, motor, and osseomuscular), whereas the neurological assessment includes several features of neurological dysfunction scored on an ascending five-point scale (0–4) and the scores are summed to obtain a total score of 56 points.

#### 2.1.3. Additional Clinical Manifestations

When copper concentration is excessive, this metal is released to the bloodstream in its free form, not bound to ceruloplasmin (NCC; non ceruloplasmin bound copper), and then, is deposited in other organs (Figure 1). The ophthalmologic features of WD include the KF ring and sunflower cataract. In the KF ring, copper is deposited within the cornea, at the level of the Descemet’s membrane, and leads to a greenish-brown opacity at the periphery of each iris. A closed KF ring would be the consequence of a long-term disease, and after treatment or liver transplant, the KF ring would decrease and even disappear; however, its reduction/disappearance does not necessarily correlate with an improvement of clinical signs. While KF ring is usually linked to WD, it can also be observed in patients suffering from other cholestatic liver diseases such as primary biliary cholangitis [13]. KF ring is detected in more than 90% of patients with neurological and/or psychiatric presentation, but only in ~50% of patients with liver symptoms, and rarely in those asymptomatic. To detect the KF ring, the American Association for the Study of the Liver disease on diagnosis and management of WD recommends ocular slit-lamp evaluation [16]. Recently, the anterior segment optical coherence tomography (AS-OCT) was reported as a diagnostic procedure to improve the detection of the KF ring; using AS-OCT, the KF ring was observed in 15 out of 29 patients with a better accuracy of 50% compared to slit-lamp examination [17]. In turn, sunflower cataract occurs in ~1.2% of patients [18]. Sunflower cataract is caused by the deposition of copper beneath the lens capsule with a central disc and radiating petal-like fonds, mimicking a sunflower [19,20]. KF ring as well as sunflower cataract rarely interfere with vision. Finally, since thinning of the total retinal nerve fiber layer (RNFL) due to axonal loss is a marker of neurodegeneration, the RNFL thickness assessed by optical coherence tomography (OCT) was investigated in WD patients, and a decrease in RNFL and macular thickness were appreciated in patients with neurological signs, who showed worse OCT parameters [21]. Nevertheless, at least for RNFL thickness, the utility of OCT as a biomarker for assessment of neurodegeneration in WD patients was not conclusive in further studies [22].

The accumulation of copper in the heart can cause conduction abnormalities, arrhythmias, cardiomyopathy, and/or sudden cardiac death [23,24]. In a study of 42 WD patients with complete echocardiographic examination, cardiac involvement was characterized by left ventricular parietal thickening with an increased prevalence of concentric left ventricular remodeling and a relatively high frequency of benign supraventricular tachycardias and extrasystolic beats [25]. Further studies revealed that cardiac manifestations are relatively frequent and can be life-threatening. In fact, cardiac arrest can occur even in liver-transplanted patients [26]. Therefore, in the diagnosis and the follow-up of WD patients, tests such as electrocardiogram, cardiac biomarkers, echocardiography, and 24-h Holter monitoring should be included [27]. By cardiac magnetic resonance-based strain analysis, a functional impairment of both ventricles was detected in 4 out of 61 WD patients, which may be helpful as a potential early sign of cardiac manifestation [28].

Excess of copper can lead to additional clinical features in other organs, including renal, osseomuscular, and endocrine manifestations, although these are rarer signs. Circulating NCC is filtered in the kidneys and excreted in urine. Nevertheless, copper accumulates in the kidney parenchyma causing acidosis, aminoaciduria and kidney stones [29]. Patients with WD may also have bone problems such as osteoporosis, spontaneous fractures and osteoarthritis, since copper can be deposited in cartilaginous and synovial joints. Finally, other rare clinical manifestations include hypoparathyroidism (due to copper deposition in the parathyroid glands), pancreatitis, amenorrhea, and recurrent miscarriages [5,30].

### 2.2. Diagnosis: Just a Matter of the Leipzig Scale?

WD clinical manifestations are diverse and not always present, potentially mimicking other diseases. In addition, none of the laboratory markers indicative of copper accumulation are 100% sensible and specific of the disease, hence the need to combine clinical findings and biochemical parameters to reach a diagnosis. The diagnosis of WD is currently based on the score developed at the 8th International Meeting on Wilson Disease in Leipzig [31], which includes clinical signs, histopathological studies, biochemical tests and genetic analysis (Table 1). A score ≥ 4 points would be confirmatory of WD, and this is already achievable if two deleterious mutations in *ATP7B* are detected segregating with the disease. Unfortunately, clinical assessment and other tests included in the Leipzig score not always yield clear findings, and genetic testing is not currently available in all settings. Below, we describe the tests commonly used in the diagnosis of WD and their suitability to assess adherence and/or compliance to therapy are discussed.

#### 2.2.1. Ceruloplasmin

Ceruloplasmin (Cp) is a copper-binding protein responsible for 75–90% of the transport of this metal in blood. When recently synthetized by hepatocytes, it is named apoceruloplasmin (Apo-Cp) and can bind six to eight copper ions [32,33], thus turning into its active form, holoceruloplasmin (Holo-Cp). Holo-Cp has ferroxidase activity, allowing the transport of ferric iron (Fe^3+^) in blood by transferrin. *ATP7B* is the main protein that “shuttles” copper to Apo-Cp, and if this event does not occur, Apo-Cp will be rapidly degraded due to its low stability.

In a comparative study using an enzymatic test and an immunologic assay for Cp measurement, the enzymatic approach based on serum Cp oxidase activity resulted to obtain a better cut-off point for predicting WD [34]. Currently, immunoassays are the most extended in clinical laboratories because they are able to detect Apo-Cp and Holo-Cp, although values can be overestimated. Reference values for immunoassays vary between 0.15 and 0.20 g/L; levels below 0.20 g/L are suggestive but if lower than 0.10 g/L, a WD diagnosis is probable (Table 1).

Cp levels tend to increase in case of inflammation, infection and hyper-estrogenemia during pregnancy or as a consequence of contraceptive treatments. In addition, WD heterozygous carriers occasionally present with abnormally reduced Cp levels. Other considerations for low Cp levels are malabsorption, fulminant hepatic failure, or aceruloplasminemia caused by mutations in *CP*. Therefore, the predictive value of Cp as a unique marker for diagnosis of WD is questionable, principally because many WD patients show bordering levels. In a recent systematic review of the Cochrane database, a threshold of 0.20 g/L was found to have a sensitivity of 77.1–99% and a specificity of 55.9–82.8%, while values < 0.1 g/L are associated with a sensitivity of 65–78.9% and specificity of 96.6–100% [35].

#### 2.2.2. Serum Copper

Total serum copper comprises NCC as well as copper contained in Holo-Cp. Total serum copper and Holo-Cp concentrations are positively correlated, and hence, decreased values of Holo-Cp are likely in WD patients. However, higher levels of Holo-Cp are detected for instance, in Wilsonian acute liver failure onset as a result of accumulated hepatic copper release to bloodstream. Moreover, increased NCC is also observed in cases of acute hepatic failure of different etiology, chronic cholestasis, and idiopathic copper toxicosis. High or normal serum copper along with low Cp indicates an increment of serum copper NCC fraction, a suggestive finding of WD. Copper belonging to the NCC fraction, sometimes not well-named as “free”, becomes harmful when deposited in tissues, mainly in liver and brain. Ultimately, the goal of chelation therapy is to reduce systemic toxic copper, thus the relevance of determining NCC in a patients’ follow-up. Normal values of NCC are <1.63 µmol/L, and in WD patients, values between 0.82–2.45 µmol/L denote treatment efficacy [36]. Usually, NCC is calculated using a formula that takes into account copper bound to Cp and the total serum copper; using SI (international system) units: NCC = total serum copper (in µmol/L) − [0.049 × Holo-Cp (in mg/L)] [33,36]. As expected, results depend on proper Holo-Cp determinations on the same sample. As a result, if Cp levels are determined by immunoassays that include the detection of Apo-Cp and Holo-Cp, NCC estimation for diagnosis and monitoring might be misleading. NCC is an indirectly calculated complex marker to monitor treatment efficacy which unfortunately shows great variability, with results that are not assessable or even negative in up to ~25% of cases [36]. New methods to directly calculate NCC independently from Cp are being developed. As an example, in France, the exchangeable copper-CuEXC (≈NCC) and the ratio of exchangeable copper (REC = CuEXC/total serum copper) are new promising tools to diagnose and monitor the disease. Indeed, a REC > 18% was found to have a sensitivity and specificity of 100% for diagnosis and family screening [37,38]. In addition, high levels of CuEXC (>2.08 µmol/L) were found to be related to extrahepatic disease with a sensibility of 85.7% and a specificity of 94.1% [39,40,41]. While used currently in most French centers, it still requires external validation.

#### 2.2.3. Urinary Copper

In treatment-naïve WD patients, urinary copper excretion (UCE), a reflection of serum NCC, is elevated, and thus, measure of copper in 24-h urinary collection is a common test for the diagnosis of WD. In symptomatic patients, values higher than 1.2 to 2 times the upper limit of normal (ULN, established on 100 µg/24 h) are registered, which may indicate possible WD. Unfortunately, method-dependent cut-offs provide a low negative predictive value (NPV) for this test, with up to 25% of individuals with proven WD (in particular children) having urinary copper levels at onset near to normality [35]. Additionally, in heterozygous WD carriers, intermediate values could be detected. To solve this, a reduction of reference levels to <40 µg/24 h for diagnosis in pediatric and asymptomatic patients was suggested [42]. Urinary copper measurement after stimulation with 1000 mg D-penicillamine is a test proposed for diagnosis in children but not in adults, with expected levels >1600 µg/24 h in WD children, yet data are inconclusive even in children [16]. Similar to total serum copper, in several conditions, such as autoimmune hepatitis, cholestasis syndromes and acute liver failure, urinary copper is elevated as a compensatory mechanism to altered biliary excretion. In the Cochrane database systematic review, a cut-off of 0.64 to 1.6 μmol/24 h (40–100 µg/24 h) used in the Leipzig criteria achieved a variable sensitivity of 50–80%, with a specificity of 75.6–98.3% [35].

UCE and serum copper are useful to check adherence and compliance to chelation therapy. UCE is more reliable than NCC to confirm adherence and success of therapy [43]. In general, levels in the range of 200–500 µg/24 h are expected for patients under D-penicillamine or trientine, although if UCE is measured after 48 h treatment cessation, near to normal values are expected (<50 µg/24 h) [44]. Levels < 75 µg/24 h assure the effectiveness of treatment for those on zinc therapy [36]. Higher excretion rates are typically observed in the first months after starting a chelation therapy to further stabilize in the long term. New tools, particularly the direct determination of NCC through the CuEXC methodology, will facilitate patient adherence and treatment monitoring but their availability is still highly limited to a few centers.

#### 2.2.4. Additional Blood Tests

Typically, persistently elevated liver enzymes, specifically aspartate aminotransferase (AST) and alanine aminotransferase (ALT), is the first disease manifestation that should alert medical specialists of a possible WD case in absence of any known etiology, particularly in young patients. Biochemical parameters, as serum alkaline phosphatase (ALP) and total bilirubin, may be informative at some point to distinguish ALF due to WD from that of different etiology. Several studies have investigated the ratio of ALP to total bilirubin for this purpose, determining that ratios <4 are suggestive of WD [45].

Once therapy is initiated, near normalization of liver function tests is typically accomplished after 1–2 years. Careful monitoring of liver enzyme levels should always be addressed in all patients. Alterations of liver enzyme levels together with inconclusive UCE and NCC results, may suggest lack of efficacy or adherence to the treatment [46].

Radioactive copper test (measurement of ^64^Cu) has demonstrated to be an additional accurate method for the diagnosis of WD [47]. ^64^Cu was intravenously injected and blood samples were drawn after 2, 24 and 48 h, and the results were expressed as ratios of serum radioactivity 24 and 48 h to 2 h, after copper administration. WD patients showed significant lower ^64^Cu ratios compared to healthy individuals and carriers of one *ATP7B* mutation with a sensitivity of 98.6% for 48 h/2 h ^64^Cu ratio [47].

Finally, an alternative technique for diagnosis of WD is the quantification of *ATP7B* peptide by immunoaffinity enrichment mass spectrometry in dried blood spot (DBS). A pilot study was carried out in thirteen WD patients; the assay precision was <10% CV, and the protein was stable for a week in DBS at room temperature [48]. Later, in a large clinical series with 216 patients, the method resulted to have a sensitivity of 91.2% and a specificity of 98.1%, aiding to solve ambiguous cases without genetic diagnosis [49].

#### 2.2.5. Liver Biopsy

Clinical practice guidelines only recommend intrahepatic copper quantification if the remaining evidence are insufficient to achieve a diagnosis, or if other liver pathology is suspected apart from WD. If other disorders are discarded, a hepatic copper content higher than 250 µg/g dry weight is considered confirmatory of WD. Other studies suggest that this threshold could be fixed in 75 µg/g dry weight for better sensibility, although a lower threshold may mean more false positive diagnoses [13]. Specific stains (rhodanine or orcein) are only useful in case of copper deposition in lysosomes, a finding rarely present. Since copper deposition across liver structures is dependent on disease stage, quantification of intrahepatic copper is not always reliable. In later stages of WD, copper deposition is often inhomogeneous, so a liver sample is usually not representative and copper concentration results are generally underestimated [16].

Early liver histological findings resemble those representatives of non-alcoholic fatty liver disease (NAFLD) and non-alcoholic steatohepatitis (NASH), comprising simple steatosis, glycogenated hepatocyte nuclei and focal necrosis. In untreated or late-diagnosed patients with advanced liver disease, copper overload and toxicity lead to inflammation, fibrosis, and eventually cirrhosis. Since liver biopsy is an invasive method, there are few studies assessing the impact of treatment on liver histology or intrahepatic copper content. Taking into account the mechanisms of action of both chelators and zinc, a dramatic decrease in deposited copper in liver is not expected, given the fixation of copper by metallothioneins and GSH in the tissue. In a study where pre- and post-treatment biopsies were compared, no significant correlation between histological findings and clinical parameters or initial presentation was observed. In 7 out of 60 WD patients under therapy, disease progression was evidenced by liver biopsies, yet no significant differences on biochemical parameters (ALT, Cp, serum copper, and UCE) and intrahepatic copper quantification were observed [50]. Similarly, in a study including 12 WD patients who underwent liver biopsy after years of treatment, disease progression determined by histological findings was independent of the increase in liver enzyme levels in serum, pointing towards a lack of correlation between biochemical and histological findings nor intrahepatic copper concentration [51]. These findings raise the question of the validity of non-invasive markers of liver disease damage (please see next paragraph).

#### 2.2.6. Imaging Evaluations: Liver and Brain Assessment

Defining the degree of liver disease at diagnosis and during chelation therapy is crucial in order to establish the most suitable treatment. Despite its invasive nature, liver biopsy remains the gold standard to determine the presence of steatosis, inflammation and fibrosis [52]. Transient elastography (TE) is an extended imaging technique, based on the measurement of tissue stiffness by ultrasounds, useful to ascertain the degree of hepatic fibrosis in several liver diseases, particularly in hepatitis C. Few studies have evaluated its performance as well as that of other non-invasive scores based on classical serum markers such as FIB-4 or APRI (AST-to-Platelet Ratio) in WD. TE solely or in combination with FIB-4 or APRI scores seems to provide an adequate positive predictive value (PPV) for diagnosis of advanced fibrosis and cirrhosis in WD patients [53,54]. Of note, the optimal threshold TE values reported in WD patients to discriminate cirrhosis (≥9.9 kpas) are notably lower than those for other liver diseases [53,55,56]. Furthermore, its reliability is higher in those recently diagnosed (<1 year) compared to those previously diagnosed with an AUC of 0.96 for the former vs 0.70 for the latter reported in a multicentric Austrian–German study that included 188 WD patients with both liver biopsy and TE (44 performed <1 year from diagnosis and 144 with >1 year after diagnosis). Based on their findings, the authors suggested that its value is possibly higher for disease staging at diagnosis and less so for disease monitoring in the long term [55], yet these findings could also be attributed to the time gap between biopsy and TE in the two groups. Recently, alternative methods for the assessment of liver fibrosis have been evaluated for WD. In a study of a cohort of 60 patients using collagen proportionate area (CPA), TE, and shear wave elastography (SWE), the authors concluded that collagen content assessment was useful; yet, single time-point elastographic liver stiffness measurements had limited applications for diagnosis of WD [57]. Liver MRI is also used to evaluate liver impairment in WD patients; the most relevant feature is the honeycomb pattern according to a retrospective study performed in a cohort of 23 adult patients, although its sensitivity seems to be limited [58].

Apart from patients with predominant neurological manifestations, clinical evaluation by a neurologist or specialist in movement disorders is recommended in all cases. Brain MRI is essential to assess presence of copper deposits and damage on basal ganglia. Common recognized abnormalities include detection of hyperintensities on T2 sequences, mainly in putamen and caudate nucleus; the “face of the giant panda” on the midbrain is considered a characteristic sign. Alterations in brain MRI at diagnosis are present in all neurological patients, in 40–75% of hepatic patients and in 20–30% of asymptomatic individuals [59]. Brain MRI to monitor recovery might be of interest, especially in cases of severe neurological impairment at onset. Interestingly, T2 signal hypointensites in basal ganglia have been systematically registered on WD patients under therapy, which suggests the deposition of paramagnetic metals [60,61]. In fact, according to post-mortem histopathological and MRI studies, this observation would correspond to the presence of copper and iron depositions in basal ganglia [62]. To assess brain MRI severity, a scale was recently validated based on acute toxicity and chronic damage subscores from predefined structures taking into account T2 hyperintensities and brain atrophy [12]. The temporal evolution of the MRI severity score and its correlation with clinical severity, assessed with the UWDRS [63], was also calculated resulting in a semiquantitaive assessment of radiological WD severity helpful for WD diagnosis, treatment monitoring, and outcome prediction [12].

Moreover, several approaches using new MRI techniques as quantitative susceptibility mapping (QSM) and susceptibility weighted imaging (SWI) have been applied to assess the presence of metal deposition on neurological and hepatic WD patients under therapy [64,65]. These techniques have demonstrated that, despite chelation therapy, iron deposition in the deep grey matter (DGM) involving basal ganglia may progressively increase, especially in neurological patients. However, long-term follow-up studies on larger cohorts are necessary to determine possible correlations between iron accumulation and brain damage, psychiatric impairment or neurological disease aggravation.

## 3. A Mendelian Disease Caused by Mutations in *ATP7B*

**Figure 2:**
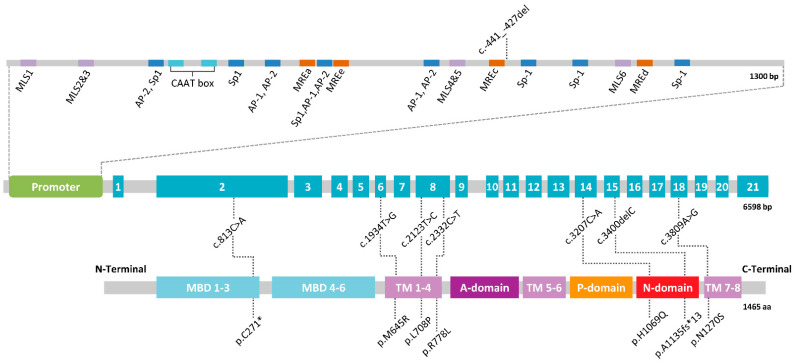
Scheme of the *ATP7B* gene with common mutations. For the promoter region, most relevant elements and transcription factor binding sites are depicted, as described in Oh et al. [69]. Abbreviations: MRE, metal response element; MLS, MRE-like sequence; MBD, metal binding domain; TM, transmembrane domain; A-domain, actuator domain; P-domain, phosphorylation domain; N-domain, nucleotide binding domain; aa, amino acids; bp, base pairs.

WD is a well-established monogenic condition caused by deleterious variants in *ATP7B* inherited in an autosomal recessive manner [66,67]. The *ATP7B* gene spans 80 kb in the chromosome 13p. The longest transcript (NM_000053) detected in liver, ranging from 7.5 to 8.5 kb, comprises 21 exons (Figure 2). Other shorter transcripts, which mainly lack exons 6, 7, 8, or 12, are present in the brain, although it is unknown if they are coding or just have a regulatory function. The promoter extends about 1.3 kb upstream from the start codon, and is characterized by four metal response elements (MREs) and six MRE-like motifs, which are *cis*-regulatory DNA sequences that specifically bind MTF-1 (metal response element-binding transcription factor-1), crucial for transcriptional induction upon heavy metal load [68]. *ATP7B* is mainly expressed in the liver and brain and significant levels of expression have been detected in the kidneys, placenta, and lungs.

*ATP7B* exhibits a high allelic heterogeneity. To date, more than 900 variants have been described in *ATP7B* (HGMD Professional version 2021.1, accessed August 2021). The vast majority are located in the coding and flanking intronic sequences throughout the entire gene, the most frequent being missense and nonsense mutations (>60%) (Table 2).

Stratified genetic analysis is recommended in order to achieve a reasonable diagnostic success rate with the aim to cover the whole *ATP7B* sequence and the different possible mutation types [70,71]. First, it is advisable to perform the study of the 21 coding exons and flanking intron sequences, since they harbor the majority (~95%) of the clinical variants (Table 2). This approach can be done by Sanger sequencing, which allows the detection of point mutations as well as small deletions and insertions. In populations in which a small number of pathological changes predominate, the prioritization of these changes, likely founder events, is a cost-effective strategy. For this, HRM (high resolution melting) techniques, customized SNP (single nucleotide polymorphism) microarray, or RFLPs (restriction-fragment-length-polymorphism) test can be used [72,73,74,75].

If no diagnosis is achieved, the possibility of large deletions or insertions must be examined by MLPA (multiplex-ligation probe amplification), in addition to sequencing the promoter. These clinical variants represent ~4% of the total known *ATP7B* mutations (Table 2). The promoter region comprises ~1.3 kb, so multiple PCR amplicons are required to cover it. Its analysis is not included in all studies, which, furthermore, does not always have the same design. The analysis is usually limited to the first 500 bp of the promoter that includes the founder deletion described in Sardinia, c.-441_-427del (Figure 2) [76]. Nevertheless, mutations placed in more distant regions have been reported as well, suggesting the convenience of a full study of the promoter sequence [70,77].

Finally, the study of the introns must be taken into account if no biallelic mutations have been detected in *ATP7B* in a patient with clinical suspicion of WD. Characterization of deep intronic mutations is a challenge, given that *ATP7B* has introns of considerable size (for instance, the intron 1 spans ~36 kb) with a high content of repetitive elements [78] that make unfeasible its analysis by Sanger sequencing. The diagnostic tools based on NGS (next generation sequencing) allow for studies of large tracts, and hence, the analysis of the entire 75 kb sequence of *ATP7B* (exons, introns, 3′ and 5′-UTR regions, and promoter) [70]. In fact, using a custom NGS tool, one deep intronic deleterious mutation was located in intron 12, c.2866-1521G>A [79]. Of note, NGS tools also allow the identification of large CNVs (copy number variants); therefore, it is an approach able to substitute the analysis using MLPA.

## 4. Wilson’s Disease Is Not That Rare

The widely accepted prevalence of WD was estimated in 1984 by Scheinberg and Sternlieb [80]: 1:30,000 (=3.3/100,000), before the responsible gene was discovered. The frequency of carriers, calculated according to Hardy–Weinberg equilibrium and full penetrance, is considered to be 1:90 [81]. As expected, the prevalence increases in closed/isolated populations, as is the case of the Canary Islands (Spain; 8.1/100,000), Sardinia (Italy; 37/100,000), or Kalymnos (Greece; 135/100,000) [72,82,83].

Many studies have intended to establish accurate values of prevalence with findings related to the strategy applied such that higher prevalence rates have been described in mutational screening studies compared to those based on public health registries (Table 3). These discrepancies are mainly due to differences between genetic and clinical prevalence. As mentioned before, diagnosis of WD is based on the Leipzig score and relies on imperfect clinical, biochemical, histological, and genetic findings. Despite technological advances, only one mutation or no mutation is identified in a relevant proportion of patients (~1–27%) [71]. Thus, Leung et al. [84] estimated a clinical prevalence of 3.3 and a genetic prevalence of 14.3 per 100,000, disparities in part explained by the contribution of several factors such as epigenetics, metabolism, incomplete penetrance and missed diagnoses. Gao et al. [85] established a clinical prevalence of 1.38 and a genetic prevalence of 12.7 per 100,000 in a meta-analysis of previous epidemiological and genetic studies, respectively. As a whole, genetic prevalence is higher highlighting that WD is possibly an underdiagnosed disease. A direct consequence is that a person may start to receive treatment late in the course of the disease when the injury is beyond the point of no return.

In view of the relative frequency of WD and the availability of a rationale therapy, it would be recommendable to include WD in the list of diseases at risk of neonatal screening. Methods based on the determination of Holo-Cp, serum copper, and UCE have limited predictive value in children. An alternative to routine biochemical tests could be the quantification of *ATP7B* protein in DBS as previously mentioned [48]. Finally, NGS has lowered the cost and time required with an improved efficacy, putting this technology within the reach of clinical routine.

## 5. Genetic Modifiers

The clinical spectrum of WD is widely extensive, with hepatic, neurological, and psychiatric manifestations, showing incomplete penetrance and variable expressivity within the same family and, even between monozygotic sibs [91,92]. Interestingly, patients carrying two deleterious mutations in *ATP7B* have been diagnosed at a very old age [93,94,95]. These cases that seem to escape Mendel’s laws of inheritance are all consequence of a missing heritability, a term coined by Prof. Brendan Maher for diseases with polygenic inheritance but time has shown that they also fit monogenic diseases [96]. Factors other than primary disease-causing mutation contribute to the clinical outcome such as genetic modifiers, epigenetics, and environmental aspects. Thus, several genetic modifiers have been suggested for WD [97].

Copper deposits lead to mitochondrial dysfunction and cause hepatic steatosis, so that genes involved in lipid metabolism may be good candidates to act as genetic modifiers, such as PNPLA3 and APOE. *PNPLA3* encodes for a lipase that hydrolyzes triglycerides, and c.444C>G (p.I148M; rs738409) was associated with an increased risk of developing steatosis in multiple disorders, from NAFLD to hepatitis B and C [98]. In a study including 98 WD patients, *PNPLA3* c.444C>G and early onset were linked to advanced steatosis, whereas hepatic copper concentration was not relevant [98]. The APOE protein is abundant in the brain as it is the main lipid transporter in cerebrospinal fluid. In a study with a cohort of WD patients homozygous for *ATP7B* p.H1069Q, Ferenci’s group concluded that the APOE genotype plays an important role in delaying the onset of neurological and hepatic symptoms [99]. However, these conclusions were not confirmed in later studies [100].

In the cell, accumulation of copper produces an increase of ROS, which triggers oxidative stress and mitochondrial dysfunction. In the *MTHFR* gene, the variants c.677C>T (rs1801133) and c.1298A>C (rs1801131) were analyzed in a cohort of 245 WD sufferers. Both polymorphisms are associated with higher levels of homocysteine in the blood which may be neurotoxic [101]. Liver functional disturbances lead to defects in the metabolism of methionine and homocysteine, which in turn generates oxidative stress. *MTHFR* c.677C>T and c.1298A>C seem to be related to age of onset and mode of presentation [102].

The abnormal deposits of iron contribute to the worsening of the clinical picture, since metabolism of iron and copper are closely related. HFE (homeostatic iron regulator) and DMT1 (divalent metal transporter 1) are related to iron metabolism. *HFE*, a gene associated with hemochromatosis, regulates endocytotic iron uptake in enterocytes: HFE competes with transferrin (Tf), a plasma iron transporter, to bind its receptor, TfR. Polymorphisms p.H63D and p.C282Y in HFE would cause a loss of TfR binding capacity, resulting in increased iron entry into the cell [9]. Initially, two cases of concomitance between variants in HFE, *ATP7B* and accumulation of iron and copper in the liver were described [103,104]. In a study of 32 patients in Sardinia, carriers of HFE p.H63D were found to have a worse response to chelator therapy and higher levels of iron in the liver [105], although this finding failed to be replicated in further studies [106,107]. Free iron in plasma, not bound to transferrin, is introduced into the cell in its reduced form (Fe^2+^) by DMT1. In a study including 108 WD patients, the *DMT1* c.396+44A>C (rs224589) variant was predominant in the patient cohort, but no phenotypic differences were observed between carriers and non-carriers [108].

COMMD1 and ATOX1 are the main *ATP7B* interactors in copper metabolism, and that is why, they have been widely studied as possible genetic modifiers of WD. Furthermore, *COMMD1* is the gene responsible for canine cuprotoxicosis in Bedlington terriers. However, genotype-phenotype correlations of variants in these two genes were controversial in WD patients [109,110,111,112,113]. In the series of 109 probands studied by Gupta et al. [112], a single case was identified with the change *COMMD1* c.521C>T (p.T174M), which is related to a greater accumulation of copper. Regarding *ATOX1*, only in one study that included 50 patients and 60 controls, the authors identified an exonic change, c.40G>A (p.G14S), in two families [114]. In silico, they determined that ATOX1 p.G14S would be located in a copper-binding motif, which may negatively interfere with the binding of ATOX1 to the MBD4 domain of *ATP7B* for copper transfer.

Finally, XIAP (X-chromosome-associated apoptosis inhibitor) was suggested as a possible genetic modifier of *ATP7B* based on its involvement in the regulation of cuprotoxicity-induced cell damage. High levels of copper were reported to promote XIAP degradation, resulting in caspase-3-mediated activation of apoptosis [115]. On the other hand, XIAP could participate in the maintenance of copper homeostasis through its interaction with COMMD1 to regulate its expression [116,117]. Weiss et al. [118] carried out association studies of known SNPs in XIAP without being able to determine a significant correlation between these, the age of onset and the clinical presentation in a series of 98 patients.

## 6. Wilson-Like: Genetic Diseases That Mimic the Wilson Phenotype

WD is clearly established as a monogenic disorder caused by *ATP7B* mutations and no additional genes seem to participate in the etiopathogenesis. However, disparate inherited diseases that mimic a Wilson-like phenotype are known. That is why, if *ATP7B* screening fails to identify a disease-causing mutation, further exhaustive genetic testing using NGS tools should be considered.

### 6.1. Congenital Disorders of Glycosylation

Congenital disorders of glycosylation (CDG) linked to mutations in *CCDC115* (MIM 616828) or *TMEM199* (MIM 616829) can display phenotypic similarities with WD and in fact, some patients suffering from a CDG have been described with an initial misdiagnosis of WD [70,119,120]. CDGs are a heterogeneous group comprising more than a hundred monogenic congenital diseases affecting multiple glycosylation pathways of proteins and lipids. CDG are classified into CDG-I type, affecting endoplasmic reticulum (ER) N-glycosylation, and CDG-II type, affecting N-glycan modification in the Golgi apparatus (GA).

Many CDGs show multisystemic clinical outcomes, which commonly affect the central nervous system. CCDC115-CDG patients present with CDG-II type serum transferrin profile related to hepatosplenomegaly, neurological affectations, elevated serum ALT and ALP levels, mild hypercholesterolemia, low serum Cp, and Wilson-like copper disturbances. On the other hand, individuals with *TMEM199* mutations exhibit clinical signs that overlap with CCDC115-CDG patients, although their symptoms seem to be milder. They present CDG-II type serum transferrin profile related to steatosis, elevated serum levels of ALT and ALP, hypercholesterolemia, low serum Cp and slightly alterations of copper metabolism; they do not develop hepatosplenomegaly or neuropsychiatric disturbances [121,122].

CCDC115 is localized in the ER-Golgi intermediate compartment (ERGIC) in hepatocytes. Its loss of function has been linked to the alteration of GA homeostasis and the inability to carry out its main functions: post-translational modifications and protein sorting and secretion. The abnormal N-glycomic profile of CCDCD115-CDG patients in serum is compatible with an overall detriment of GA function [122]. Concerning copper abnormalities, dysregulation of GA could be responsible for them too. In normal conditions, *ATP7B* is located in the trans-Golgi network (TGN) of hepatocytes where it performs two core functions: to incorporate copper into apoceruloplasmin and to excrete excessive copper in the bile. Raised copper intracellular levels cause the migration of *ATP7B* from trans-Golgi location to a post-Golgi vesicular compartment of the bile canaliculus membrane, a critical biliary copper excretion pathway. Alteration in GA could induce an incorrect distribution of *ATP7B* and a decrease in its activity [119]. In addition, a recent study has shown that several COG (conserved oligomeric Golgi complex) subunits- involved in Golgi homeostasis and glycosylation- are found within the *ATP7A* interactome, and hence, Golgi homeostasis, glycosylation and intracellular copper homeostasis are linked [123]. Moreover, autophagy may be involved in the CCDC115-CDG-associated disease mechanism, since the development of steatosis and liver fibrosis has been connected to a defective autophagy [124,125].

*TMEM199* also plays a role in the homeostasis of GA. In yeast, Vma22p protein (the TMEM199 homologous) stabilizes the V0 domain during V-ATPase (vacuolar-type ATPase) assembly. Human V-ATPase participates in the acidification of endosomes of the secretory pathway, including GA. Taking this into account, failures to acidify GA may dysregulate its function and disrupt the glycosylation machinery [120,121].

### 6.2. Progressive Familial Intrahepatic Cholestasis

Progressive familial intrahepatic cholestasis (PFIC) is a heterogenous group of autosomal recessive liver disorders characterized by defective bile transport. One of its early onset forms, PFIC3 (MIM 602347), is caused by mutations in the *ABCB4* gene that encodes a class III multidrug resistance P-glycoprotein. This protein mediates the translocation of phosphatidylcholine across the canalicular membrane of hepatocytes and its decreased activity or lack of function results in impaired phospholipid secretion. Damage to the biliary epithelium is caused by a continuous exposure to bile acids, whose detergent effects are not buffered by phospholipids, triggering cholestasis, hepatic fibrosis, cirrhosis, and hepatocellular failure [126]. If PFIC3 is not diagnosed early, chronic cholestasis can lead to elevated hepatic copper concentration and increased urine copper excretion, symptoms that overlap the current diagnosis for WD. A few patients have been reported who were initially considered as WD patients but were eventually diagnosed with PFIC3 owing to the identification of *ABCB4* mutations [127,128].

### 6.3. Aceruloplasminaemia

Aceruloplasminaemia (MIM 604290) is an autosomal recessive disorder caused by mutations in the *CP* gene, which encodes ceruloplasmin, one of the most important plasma ferroxidases in mammalians. Cp has two isoforms, the soluble form and the membrane-bound form (GPI-Cp) that is responsible for iron homeostasis in the brain. In the absence of GPI-Cp, ferrous iron (Fe^2+^) is accumulated in astrocytes, resulting in oxidative damage and a decreased protective function of neurons. Neuropsychiatric manifestations may include dementia, cognitive deficit, behavior alterations, tremor, chorea, cerebellar ataxia, nystagmus, dysarthria, rigidity and akinesia. Additional symptoms such as retinal degeneration, diabetes mellitus, and microcytic anemia are the result of iron deposits [129,130]. Although a low level of Cp in serum is associated with WD, normal concentration of hepatic copper and in urinary copper excretion can be discriminant indicators between both syndromes, WD and aceruloplasminaemia.

### 6.4. Menkes Disease

Menkes disease (MNK; MIM 309400) is an X-linked syndrome caused by mutations in *ATP7A*. *ATP7A*, as well as *ATP7B*, is a transmembrane copper transporter localized in the TGN and its dysfunction turns into deficient copper transfer to the secretory pathway. The pathophysiology can be explained by the loss of function of copper-dependent enzymes in the whole organism.

Allelic disorders of MNK disease are occipital horn syndrome (OHS; MIM 3040150) and X-linked distal spinal muscular atrophy 3 (SMAX3; MIM 300489). These three entities are best described as a clinical continuum spectrum from severe to mild forms. MNK is characterized by epilepsy, progressive neurodegeneration, conjunctive tissue abnormalities, a distinctive hair appearance and short lifespan (age of death < 3 years) [131]. A case of an unusual phenotype resembling WD was reported by Bansagi et al. [132]. The patient displayed complex neuromuscular signs of bilateral nystagmus, dysarthria, spastic tetraparesis, dystonia, ataxia, and axonal motor neuropathy, together with T2 hyperintensity in the globus pallidus on brain MRI, which is a feature associated with WD.

### 6.5. MEDNIK Syndrome

MEDNIK syndrome (mental retardation, enteropathy, deafness, neuropathy, ichthyosis and keratoderma; MIM 609313) is a rare neurocutaneous disorder with multisystem involvement, inherited in an autosomal recessive fashion. Patients display a perplexing picture of MNK disease and WD. Some neurological, skeletal, and cutaneous signs, as well as low copper and Cp levels in plasma, resemble MNK but in a milder form. On the other hand, hepatic damage associated to tissue copper accumulation, increased urinary copper excretion and bilateral T2 hyperintensity of basal ganglia are typical clinical manifestations of WD [133].

MEDNIK syndrome is caused by *AP1S1* mutations. *AP1S1* encodes σ1A, the small subunit of the adaptor protein 1 complex (AP-1), which plays an essential role in clathrin-coated vesicle assembly, protein sorting and regulation of vesicular trafficking between the TGN, endosomes and plasma membrane. *ATP7A* needs clathrin and AP-2 for its endocytosis and, in addition, AP-1 is required for the migration of *ATP7A* to the TGN. Dysfunction of AP-1 results in the arrest of *ATP7A* in small vesicles. The disruption in the traffic of other clathrin-dependent proteins may explain some of the diverse clinical manifestations. In addition, although the disease mechanisms underlying MEDNIK syndrome are not completely understood, there is growing evidence linking AP-1 with neuronal function and the modulation of pigmentation [133]. Moreover, the MEDNIK syndrome is an adaptinopathy caused by dysregulation of copper homeostasis. AP-1 mediates intracellular trafficking of *ATP7A* and *ATP7B*. Alterations in AP-1 may result in impaired targeting of both ATPases in the TGN and its mislocalization along the endocytic pathway. As a consequence, copper excess would not be properly excreted through bile canalicular membrane [134]. Due to its physiopathological resemblance to WD, zinc therapy is proposed to MEDNIK patients in order to reduce copper overload [133].

### 6.6. Alagille Syndrome

Alagille syndrome (ALGS; MIM 118450) is a multisystem disorder caused by mutations in *JAG1* or *NOTCH2* genes transmitted in an autosomal dominant way. ALGS is characterized by the scarcity of bile ducts in liver biopsy and, at least, the presence of three of five clinical features: cholestasis, congenital heart defects, ocular abnormalities, bone anomalies and characteristic facies. Renal disease, development, and growth retardation may occur less frequently.

Nearly 400 mutations have been annotated for *JAG1* implicated in ALGS, while cases owing to *NOTCH2* mutations are less frequent. *JAG1* encodes Jagged-1 protein, a ligand of the Notch-1 family of transmembrane receptors, which are involved in signaling pathways related to cell fate determination during embryogenesis. Overall, there are no clear genotype-phenotype correlations for this syndrome and its high variable expressivity and incomplete penetrance are well recognized [135,136]. Amson et al. [137] reported two siblings who both presented with liver disease but were carriers of two different genetic alterations. The index case was diagnosed with ALGS, and molecular analysis showed that he was a carrier of a *de novo* duplication in *JAG1* and a mutation in *ATP7B*. His brother was diagnosed with WD and resulted to be a compound heterozygous for *ATP7B* mutations. The index case had abnormal liver function, but he had no bile duct paucity on liver biopsy. So far, the question whether the *ATP7B* variant contributes to the clinical outcome in the index case remains unanswered.

### 6.7. Idiopathic Copper Toxicosis

Idiopathic copper toxicosis (ICP) is referred to a type of infancy and childhood liver disease with abnormal copper accumulation whose etiology is poorly understood [138,139]. Symptoms can include hepatosplenomegaly and abdominal distention, occasionally accompanied by ascites, lethargy, malaise, fever, anemia and, rarely, recurrent jaundice. The underlying liver affections are characterized by elevated hepatic copper concentration, destruction of the normal liver architecture (marked fibrosis and mild inflammatory infiltrate), ballooning degeneration of hepatocytes and abundance of Mallory bodies. Serum copper and ceruloplasmin concentrations are normal or slightly elevated so they can provide useful information to dismiss WD diagnosis. It has been proposed that the origin of ICP is caused by a combination of autosomal recessive defects in copper metabolism and the exposure to abnormally high dietary copper. Although the candidate gene for ICT has not been identified yet. Harada et al. [140] reported a patient who suffered from progressive hepatic damage, even after chelation therapy and successful removal of copper excess. This phenomenon suggests that, besides copper accumulation, additional unidentified mechanisms involved in developing cholestasis and inflammation are probably present.

## 7. The Need for Biomarkers for a Better Diagnosis

### 7.1. Biomarkers in WD and Related Clinical Phenotypes

The prognosis of patients with WD depends on the degree of hepatic and neurological involvement at time of diagnosis and therapeutic compliance. Indeed, early diagnosis and treatment are the keystones of successful management of WD patients. The main impediment to a good vital prognosis is the failure to make a timely diagnosis, generally conditioned by a scarce knowledge of this disease due to its low prevalence, the heterogeneous clinical presentation and the absence of a diagnostic test that allows an irrefutable diagnosis. Long-term therapy aims to avoid copper overload. Routine laboratory tests used to monitor treatment efficacy and compliance include liver enzymes, serum Cp, serum copper, and 24 h urinary copper excretion Neurological (including KF ring disappearance) and liver improvement are the first clinical indicators of treatment efficacy. Laboratory tests complemented with imaging such as brain MRI or TE are helpful to confirm treatment efficacy and compliance [52,60]. Unfortunately, as mentioned before, the reliability of TE to assess fibrosis regression is suboptimal, highlighting the need to identify non-invasive biomarkers for monitoring new treatment options and to identify potential therapeutic targets. On this subject, international agencies including the European Medicines Agency (EMA) and the U.S. Food and Drug Administration (FDA) States expressed the necessity of integrating research on biomarkers and developing new treatments.

As previously described the CuEXC assay, which calculates the percentage of exchangeable to total serum copper, REC (relative exchangeable copper), seems to be the most accurate measurement of copper overload. In two murine models, Long-Evans Cinnamon (LEC) rat and *Atp7b*^-/-^ mice, a correlation between CuEXC levels and degree of liver injury was observed and the ratio between CuEXC and total serum copper was found to be a feasible marker to distinguish them from a healthy group regardless of liver disease stage [41]. Several authors assessed measurements of CuEXC and total serum copper for diagnosis and to monitor chelation therapy in WD patients. In a series of 48 WD patients, Poujois et al. [39] reported significant incremented levels of CuEXC in neurologically impaired individuals correlating with disease UWDRS severity scale [63], compared to hepatic patients without signs of acute liver failure (ALF). Moreover, Guillaud et al. [40] assessed CuEXC and total serum copper measurements for monitoring chelation therapy. Patients with poor adherence and/or compliance to therapy showed increased levels of CuEXC and ALT/AST. In sum, CuEXC determination seems to be a reliable test useful for monitoring disease progression and compliance in WD patients.

Copper release from hepatocytes to circulation and its accumulation in tissues induce oxidative stress and triggers an inflammatory response, which contributes to WD pathogenesis. In patients with neurological symptoms, increased serum levels of lipid peroxidation and reactive oxygen species (ROS) markers, such as malondialdehyde (MDA) and glutamate, along with cytokines (IL6, IL8, IL10, and TNF-α) have been described [141]. In addition, a profile of polyunsaturated fatty acids named oxylipins was differentially detected in WD patients, which act as mediators in oxidative stress injury and inflammation [142]. Consequently, these analytes could potentially be used to assess the progression of neurological symptoms.

Under the assumption that macrophage activation contributes to the development of fibrosis, inflammation and portal hypertension in liver, association of sCD163 (macrophage activation marker soluble) with acute liver disease severity in WD was investigated. Preliminary findings showed that sCD163 had a major presence and high correlation with liver function markers (ALT, AST and GGT) in WD patients with ALF or cirrhosis [143]. Proteomic studies in biofluids allowed the identification of alternative inflammation markers in WD patients. Analysis of plasma proteomic profile revealed abundance of fibrinogen in WD patients in contrast to other patients with different pathologies also leading to liver fibrosis. Fibrinogen is an acute-phase reactant synthetized by the liver that increases in response to inflammation. The study of the microbiome is a dynamic research field that has proved its implication in liver homeostasis [144] and in the effect of ingested copper on microbial activity [145]. Gut microbiota influence several biological processes such as the immune response, digestion and metabolism through host-symbiont interactions [146]. Disturbances of host homeostasis causes translocation of gut microbiota metabolites and components to the liver and other organs inducing inflammation [147]. In WD patients, a decrease in gut bacteria related to impaired immune response was observed [148]. In this regard, the deregulation of inflammatory cytokines in WD patients may be associated with a severity of hepatic and neurologic clinical manifestations [149]. Proteomic analyses comparing asymptomatic WD and control individuals showed that complement component C3 and B, and α2-macroglobulin belonging to the innate immune system and implicated in oxidative stress and inflammation are good biomarkers for early WD stages [150]. Recently, gut dysbiosis in WD patients has been settled on a diminished microbial ecosystem with loss of bacterial diversity engaged in transcription factors and ATP-dependent transporters (ABC; ATP-binding cassette) [151]. Of interest for its implication in the development of hepatic steatosis and inflammation, dysregulation of choline metabolism has been described in WD patients and in the tx-j mice model [152]. The integrative microbiome and metabolome framework is an amazing research field searching for more specific metabolites acting as messengers between the microbiota and the immune system.

### 7.2. Potential of Circulating Micrornas as Biomarkers in WD

Circulating microRNAs (miRNAs) in biofluids are broadly investigated as non-invasive markers for diagnosis and prognosis of a plethora of diseases, as neoplasia, hepatic, cardiovascular, autoimmune and neurodegenerative disorders, even viral infections, to name a few. miRNAs are evolutionary conserved non-coding RNA molecules of 16–28 nucleotides, the function of which is regulating gene expression by base complementary binding to intronic or 3′ UTR regions of mRNA target genes. miRNAs act inhibiting the expression of genes in the parent cell, and they are also released as a consequence of tissue injury, apoptosis, and necrosis events, or as a part of a communication mechanism between cells, tissues and organs. Moreover, miRNAs expression is cell and tissue-specific and their circulating measured levels correlate with response to treatment and disease progression, being detectable in early stages [153].

To date, more than 2000 miRNAs are known, localized in coding and non-coding (intergenic and intronic) regions and modulating the expression of up to 60% of genes encoding proteins implicated in cell cycle control, differentiation, proliferation, apoptosis and metabolism [154]. The liver is an organ with a high regenerative capacity involved in vital metabolic processes and that is why miRNAs are essential for its regulation. Alterations of miRNAs expression pattern in liver and variations of its levels in biofluids, mainly serum and plasma, have been associated with different hepatic clinical features such as steatosis, hepatitis, cirrhosis, and hepatocellular carcinoma. miR-122 is the most abundant, specific adult-liver expressed miRNA, which represents more than 70% of liver miRNome. This miRNA plays a key role in metabolic functions along with hepatocyte homeostasis, differentiation and development processes [155]. As far as WD is concerned, Siaj et al. [156] evaluated serum miR-122 levels and liver function markers (ALT, AST and bilirubin) in the LEC rat model for WD with a high copper diet to induce liver damage. Circulating miR-122 levels increased significantly faster than other liver function markers, reaching a maximum value at fulminant hepatitis onset. Furthermore, in rats undergoing hepatocyte cell-based therapy, circulating miR-122 levels rapidly decreased down to baseline levels.

## 8. Conclusions

There are multiple diagnostic, therapeutic, and monitoring tools for patients suffering from WD. While many patients are diagnosed and adequately assessed with current available methods, diagnosis and monitoring have revealed to be more challenging in other patients stuck in a diagnostic uncertainty characterized by borderline ceruloplasmin levels, inconclusive genetic findings and unclear clinical phenotypes. Patient prognosis is based on early diagnosis and adequate long-life therapy. Delay in diagnosis, and, therefore, in starting decoppering treatment can result in disastrous consequences. Technological advances in genetics tests, neuroimaging, characterization of biomarkers, and advanced therapies may provide confidence for a more accurate detection and management of WD in coming years.

## Figures and Tables

**Table 1:** Diagnostic scoring system for Wilson’s disease.

Test	Parameter	Score
Kayser-Fleischer ring	Present	2
	Absent	0
Neurological symptoms	Severe	2
	Mild	1
	Absent	0
Serum ceruloplasmin	Normal (>0.2 g/L)	0
	0.1–0.2 g/L	1
	<0.1 g/L	2
Coombs-negative hemolytic anemia	Present	1
	Absent	0
Liver copper (in the absence of cholestasis)	>250 µg (>4 µmol) g^−1^ dry weight	2
	50–249 µg (0.8–4 µmol) g^−1^	1
	Normal: <50 µg (<0.8 µmol) g^−1^	−1
	Rhodanine-positive granules	1
Urinary copper (in the absence of acute hepatitis)	Normal	0
	1–2 × ULN	1
	>2 × ULN	2
	Normal but >5 × ULN after D-penicillamine	2
Mutation analysis of *ATP7B*	Biallelic deleterious variants	4
	One deleterious variant	1
	No mutation detected	0
Total score	Evaluation	
≥4	Diagnosis established	
3	Diagnosis possible; more tests needed	
≤2	Diagnosis very unlikely	

ULN: upper limit of normal.

**Table 2:** Types of Known Variants in *ATP7B* and its Frequency ^1^.

Type of Variant	No. Mutations	Frequency (%)
Regulatory sequences	12	1.28
Splicing site	77	8.21
Missense and nonsense mutations	572	60.98
Small deletions	158	16.84
Small insertions	80	8.53
Indels	12	1.28
Gross deletions	26	2.77
Deep intronic	1	0.11

^1^ Data obtained from the HGMD Professional version 2021.1.

**Table 3:** Prevalence of Wilson’s disease in several populations.

Source	Prevalence	Country	Reference
Public health registries	1.81/100,000	Taiwan	[86]
	1.50/100,000	France	[87]
	1.64/100,000	Spain	[88]
Mutational screening in a population	14.28/100,000	United Kingdom	[89]
	13.22/100,000	South Korea	[74]
	25/100,000	France	[90]

## Data Availability

Not applicable.
